# Suspension TRAPping Filter (sTRAP) Sample Preparation for Quantitative Proteomics in the Low µg Input Range Using a Plasmid DNA Micro-Spin Column: Analysis of the Hippocampus from the 5xFAD Alzheimer’s Disease Mouse Model

**DOI:** 10.3390/cells12091242

**Published:** 2023-04-25

**Authors:** Evangelia Thanou, Frank Koopmans, Débora Pita-Illobre, Remco V. Klaassen, Berna Özer, Ioannis Charalampopoulos, August B. Smit, Ka Wan Li

**Affiliations:** 1 Department of Molecular and Cellular Neurobiology, Center for Neurogenomics and Cognitive Research, Amsterdam Neuroscience, Vrije Universiteit Amsterdam, De Boelelaan 1085, 1081 HV Amsterdam, The Netherlands; 2 Pharmacology Department, Faculty of Medicine, University of Crete, 71003 Heraklion, Greece; 3 Institute of Molecular Biology and Biotechnology, Foundation for Research and Technology Hellas, 71003 Heraklion, Greece

**Keywords:** sTRAP, micro-spin column, Alzheimer’s disease, 5xFAD mouse model, proteomics

## Abstract

Suspension TRAPping filter (sTRAP) is an attractive sample preparation method for proteomics studies. The sTRAP protocol uses 5% SDS that maximizes protein solubilization. Proteins are trapped on a borosilicate glass membrane filter, where SDS is subsequently removed from the filter. After trypsin digestion, peptides are analyzed directly by LC-MS. Here, we demonstrated the use of a low-cost plasmid DNA micro-spin column for the sTRAP sample preparation of a dilution series of a synapse-enriched sample with a range of 10–0.3 µg. With 120 ng tryptic peptides loaded onto the Evosep LC system coupled to timsTOF Pro 2 mass spectrometer, we identified 5700 protein groups with 4% coefficient of variation (CoV). Comparing other sample preparation protocols, such as the in-gel digestion and the commercial Protifi S-TRAP with the plasmid DNA micro-spin column, the last is superior in both protein and peptide identification numbers and CoV. We applied sTRAP for the analysis of the hippocampal proteome from the 5xFAD mouse model of Alzheimer’s disease and their wildtype littermates, and revealed 121 up- and 54 down-regulated proteins. Protein changes in the mutant mice point to the alteration of processes related to the immune system and Amyloid aggregation, which correlates well with the known major Alzheimer’s-disease-related pathology. Data are available via ProteomeXchange with the identifier PXD041045.

## 1. Introduction

Bottom-up proteomics is the method of choice for the study of global protein expression in tissues and cells, which may yield insights into disease mechanisms or physiological processes of interest. A typical proteomics workflow consists of sample preparation, preferably a complete solubilization and denaturation of proteins by the use of detergents or chaotropic reagents, enzymatic digestion predominantly by trypsin that cleaves proteins at the C-terminal of Arg and Lys, liquid chromatography (LC)-mass spectrometry (MS) of the tryptic peptides, database search and downstream analysis of the acquired MS data.

Popular sample preparation protocols for proteomics analysis include the use of in-gel digestion [1,2], filter-aided sample preparation [3,4] (FASP), single-pot solid-phase-enhanced sample-preparation [5,6] (SP3), and a suspension TRAPping filter [7,8] (sTRAP), among others [9,10,11]. sTRAP is particularly attractive because of its relatively short and simple procedure with good protein recovery, effective removal of detergents, and the possibility to implement in high throughput workflows [12]. In the sTRAP protocol, the SDS-solubilized proteins form a fine suspension in methanolic solution and are trapped within a stack of borosilicate glass membrane filters. After repeated washes to remove SDS and other small contaminants, proteins are digested on filter and the resulting peptides are ready for LC-MS analysis.

The sTRAP spin column is commercially available, but relatively expensive [10] compared to materials required for other protocols. This may hinder its wider application, especially for high throughput proteomics studies. In 2019, Lin et al. [7] detailed a low-cost protocol to self-pack sTRAP filter units with a glass fiber insert. Interestingly, plasmid DNA mini-prep spin columns are packed with a similar type of glass fiber, and in principle could be used for sTRAP. Plasmid DNA spin columns are low-cost and widely available from many vendors. It is expected that the commercial product should have a more consistent physical property than a self-packed unit, and would therefore introduce less technical variation.

Alzheimer’s disease (AD) is a common age-related neurodegenerative disorder [13]. It is characterized by two main pathologic hallmarks, the Aβ plagues and MAPT tangles that are initially present in the cortex and the hippocampus, respectively, and spread to other brain regions as the disease progresses. Several mouse models have been developed to gain better understanding of the underlying molecular mechanisms leading to the AD pathology [14]. Among them, the 5xFAD transgenic mouse model has been widely used. This mouse model overexpresses human *APP* with three FAD mutations [the Swedish (K670N, M671L), Florida (I716V), and London (V7171) mutations] and human *PSEN1* with two FAD mutations (M146L and L286V) [15]. A recent deep phenotypic study [16] indicates that the 5xFAD model recapitulates certain aspects of human AD.

In this study, we performed a comprehensive analysis of plasmid DNA micro-spin column-based sTRAP proteomics to examine its reproducibility and protein recovery, focusing on protein quantities relevant for bio-medical samples that are often available at low to sub-µg levels. We used the current advanced LC-MS setup comprising of the Evosep-TimsTOF Pro 2 with a gradient of 40 min for the analysis. In the cases of 5–10 µg protein inputs for sTRAP sample preparation, the use of 120 ng of the resulting tryptic peptides for LC-MS resulted in the identification of about 5700 protein groups with CoV of 4%. For the lower input amounts of 0.6 and 0.3 µg, the CoV increased to about 6–8%, and the identified protein groups decreased to <5000. We then compared the plasmid DNA micro-spin column-based sTRAP with the commercial Protifi S-TRAP column and the in-gel digestion protocol for sample preparation. Both the plasmid DNA micro-spin column and the Protifi column were superior to the in-gel digestion protocol regarding the number of proteins and peptides detected, as well as the CoV. The plasmid DNA micro-spin column appears to perform slightly better than the Protifi column. We then applied this protocol to examine the hippocampal proteome differences between the 5xFAD mouse model of AD and its wildtype littermates. Protein changes in the mutant mice point to the alteration of processes related to the immune system and Amyloid aggregation, which correlates well with the known major AD-related pathology.

## 2. Materials and Methods

### 2.1. Animals

For all analyses, 14-week-old wildtype and 5xFAD mice were used. All 13 mice used (one for the testing of the sample preparation protocols and the LC-MS system, and 12 for the analysis of 5xFAD mice) were males and housed in a pathogen-free facility at room temperature under a 12 h/12 h light/dark cycle. Animals were allowed ad libitum access to water and standard pelleted mouse chow (complete feed for MICE and RATS, pellet 12 mm, Mucedola, Milan, Italy). The protocols used were approved by the Veterinary Directorate of Prefecture of Heraklion, Crete and were carried out in compliance with Greek government guidelines and the guidelines of the ethics committee.

### 2.2. Sample Homogenization

Hippocampi were dissected and stored at −80 °C until the preparation of P2 fraction took place. Individual hippocampi were homogenized in 1 mL 5 mM HEPES/NaOH pH 7.4, 0.32 M Sucrose and Protease Inhibitor cocktail (Roche, one tablet was added in 50 mL homogenization buffer, and 2 mL of this buffer was used for the homogenization of each individual hippocampus), using a tissue homogenizer in 900 rpm and 12 strokes (de homgen^plus^, Schuett biotec. Göttingen, Germany). The extract was centrifuged at 1000× *g* for 10 min at 4 °C. The supernatants were transferred to new tubes and centrifuged at 25,000× *g* for 40 min at 4 °C. The pellet, P2 fraction, was resuspended in the homogenization buffer. Protein concentration was determined using the Bradford assay.

### 2.3. sTRAP Protocol for Sample Preparation

For each sample, 10 μg of protein, or the amount otherwise stated in the serial dilution experiments, were transferred into Eppendorf tubes and dried. Each sample was dissolved in 50 µL 5% SDS buffer containing 100 mM Tris, pH 8 and 2 µL tris(2-carboxyethyl) phosphine (50 mM), and incubated at 65 °C for 30 min in a ThermoMixer (Eppendorf, Hamburg, Germany) for the reduction of disulfide bonds. A total of 2 µL methyl methanethiosulfonate (200 mM) was then added and incubated for 5 min at room temperature to derivatize the reduced sulfhydryls. After mixing with 5 µL 12% phosphoric acid and 400 µL washing buffer (90% methanol and 10% 1 M Tris, pH 8), the sample was transferred to a plasmid DNA micro-spin column (HiPure from Magen Biotechnology, Guangzhou, China). The micro-spin column was washed 4 times in 400 µL washing buffer by centrifugation at 1400× *g* for 1–2 min. A total of 0.45 µg Trypsin/Lys-C (Promega, Madison, WI, USA) in 45 µL 50 mM NH_4_HCO_3_ was added to the micro-spin column and incubated at 37 °C overnight in a humidified incubator. The micro-spin column was centrifuged for 1 min at 1400× *g.* A total of 100 µL 0.1% formic acid was added to the micro-spin column and centrifuged for 2 min. A total 100 µL 0.1% formic acid in acetonitrile was added and centrifuged again for 2 min. Eluents from the micro-spin column in these three centrifugation steps were collected together and speed vac dried.

### 2.4. Sample Preparation Protocols for Commercial sTRAP Column and In-Gel Digestion

The commercial sTRAP column was obtained from Protifi (Prod# CO_2_-micro-80, New York, NY, USA). For the Protifi S-TRAP workflow, all trapping and washing steps were performed as outlined by the manufacturer’s S-Trap™ micro-spin column digestion protocol [8]. Protein digestion was performed as stated above.

The in-gel digestion procedure was performed as outlined before [1,2] using a 12-well 10% SurePAGE gel (Genscript, Piscataway, NJ, USA). Protein digestion was performed overnight at 37 °C using 0.5 µg Trypsin/Lys-C (Promega) in 125 µL 50 mM NH_4_HCO_3_. Eluted peptides were transferred to LoBind Eppendorf tubes, dried by SpeedVac and stored at −80 °C.

### 2.5. LC-MS analysis

Each sample of digest was redissolved in 100 µL 0.1% formic acid and the peptide solution was loaded onto an Evotip Pure (Evosep, Odense C, Denmark) and run on a 15 cm × 150 µm reverse-phase column packed with 1.5 µm C18-beads (EV1137 from EvoSep) using the Evosep One LC system (Evosep) with the 30 samples per day program. Peptides were electro-sprayed into the TimsTOF Pro 2 mass spectrometer connected to a 20 µm ID ZDV emitter (Bruker Daltonics, Bremen, Germany). The MS was operated with the following settings; Scan range 100–1700 *m*/*z*, ion mobility 0.6 to 1.6 Vs/cm^2^, ramp time 100 ms, accumulation time 100 ms, and collision energy decreasing linearly with the inverse precursor ion mobility from 59 eV at 1.6 Vs/cm^2^ to 20 eV at 0.6 Vs/cm^2^. For the operating in diaPASEF [17] mode, each cycle took 1.8 s and consisted of 1 MS1 full scan and 16 dia-PASEF scans. Each dia-PASEF scan contained two dia-PASEF isolation windows, in total covering 400–1201 *m*/*z* (1 Th window overlap) and ion mobility 0.6 to 1.43 Vs/cm^2^. Ion mobility was auto calibrated at the start of each sample (filter application of calibration standard with *m*/*z*, 1/KO: 622.0290, 0.9917; 922.0098, 1.1984; 1221.9906, 1.3934).

### 2.6. Data Analysis

DIA-NN 1.8.1 [18] was used for the database search. Raw diaPASEF data from the TimsTOF Pro 2 were searched with a virtual spectral library generated from the mouse fasta file (UP000000589_10090.fasta) by DIA-NN. Deep-learning-based spectra were activated. Protein inference was set to isoform and cross-run normalization was activated. The precursor charge range was 2–4. A fixed modification of UniMod: 39, 45.987721, C was used, which represents the MMTS modification on the cysteine residue. All other settings used were the default settings.

For the downstream analysis, we used MS-DAP (version beta 0.2.5.1 [19]) for quality control and candidate discovery. Differential abundance analysis between groups was performed on log-transformed protein abundances. Empirical Bayes moderated t-statistics with multiple testing correction by false discovery rate (FDR) ≤ 0.05, as implemented by the DEqMS function, was used as previously described [20]. ShinyGO [21] was used for GO analysis. String (version 11.5 [22]) was used to construct the functional protein association network. The mass spectrometry proteomics data were deposited to the ProteomeXchange Consortium via the PRIDE [23] partner repository with the dataset identifier PXD041045.

## 3. Results

In the present study, we first demonstrated that the use of a plasmid DNA micro-spin column as an sTRAP filter for sample preparation produces good data quality in technical replications and serial dilution experiments down to low protein input of about 0.3 µg. We compared it with other sample preparation protocols, as the commercially available columns for sTRAP and the in-gel digestion, and then applied the protocol to analyze the hippocampal extract from a mouse model of AD.

### 3.1. Plasmid DNA Micro-Spin Column Provides Excellent Filter for sTRAP Sample Preparation

To examine whether the commercial plasmid DNA micro-spin column is applicable for sTRAP, we processed the synaptosome-enriched fraction (P2) in the micro spin-column (Magen Biotechnology, Guangzhou, China, catalog number HiPure C13011 containing glass fiber filter membrane GF/F). The P2 fraction (10 µg) was solubilized in 5% SDS buffer, and mixed with methanolic solution to cause the formation of a fine suspension. With this low protein amount, the fine suspension was not visible, and the solution remained clear. The whole procedure from the loading of the sample to the addition of trypsin took <30 min. The conventional trypsin digestion protocol was followed with overnight incubation at 37 °C.

**Figure 1:**
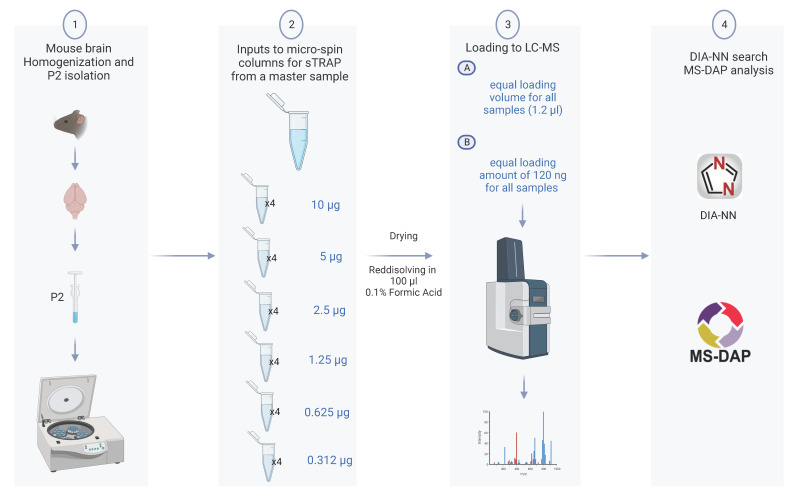
Experimental design of the sTRAP proteomics analysis to reveal the reproducibility and depth of analysis. (**1**) A P2 fraction enriched in synapse was prepared from a mouse brain. (**2**) A single P2 sample was serial diluted from 10 µg down to 0.3 µg, with four technical replicates for each dilution, and processed in the plasmid DNA micro-spin column. (**3**) Tryptic peptides from each sample dilution was run twice in the LC-MS with either a constant loading volume of 1.2% (A), or a constant protein amount of 120 ng (B). (**4**) Raw data were searched with DIA-NN, and the downstream analysis including quality control and statistical test with MS-DAP (created with BioRender.com).

To assess the reproducibility and the trapping capacity of the micro-spin column for low protein amount, we prepared a dilution series from a single P2 fraction and examined how the protein amount loaded onto the micro-spin column affected the subsequent proteomics analysis. We performed two series of LC-MS measurements from each sample keeping constant either (A) the loading volume corresponding to 1.2% of the samples or (B) the protein amount of 120 ng for all samples. The study design is shown in Figure 1.

**Figure 2:**
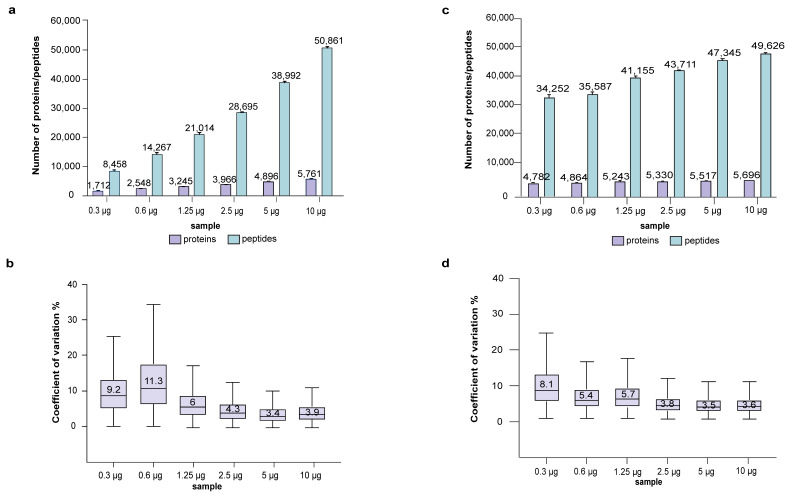
Performance of plasmid DNA micro-spin column for sTRAP proteomics. (**a**) The number of proteins/peptides identified and (**b**) boxplots showing the CoV from the serial diluted samples using 1.2% of the eluted peptides, (**c**) Proteins/peptides identified and (**d**) boxplots showing the CoV using 120 ng of the eluted peptides. X-axis shows the original input amounts that were serially diluted from the 10 µg sample. Four technical replicates were performed for each group.

In the first experiment, 1.2% of the tryptic peptides eluted from the micro-spin column were analyzed by LC-MS (Figure 1), the amount of protein loaded into the Evotip corresponded to 120 ng, 60 ng, 30 ng, 15 ng, 7.5 ng and 3.75 ng for the six serial diluted samples, with four technical replicates per dilution. Figure 2a,b shows the numbers of protein/peptide identified and CoV from each dilution. Approximately 5700 protein groups with a median CoV of 3.9% were obtained from the high load of 120 ng. A decrease in loading amount was accompanied by the corresponding decrease in the number of identified proteins/peptides, while the CoV increased to around 10% for the lowest loading of 3.75–7.5 ng. This increase in CoV is a result of the combined variation occurring in the LC-MS and the sTRAP protocol; it is expected that a lower amount injected into LC-MS would yield a higher CoV.

All existing sample preparation protocols incur some sample loss, and in certain protocols the loss is particularly acute for low sample amounts. To assess the recovery of peptides from a decreasing amount of protein loaded on the micro-spin column, we adjusted the volume of the tryptic peptides to an equivalent of 120 ng for LC-MS analysis (Figure 2c,d, Table S1). The group of 10 µg loaded on the micro-spin column yielded about 5700 protein groups with a median CoV of 3.6%, which corresponds well to the previous results (Figure 2a,b, Table S1). The decreased loading on the micro-spin column incurred some loss in identified proteins/peptides and an increase in CoV. For those at the lower end of loading (300–600 ng on spin column), we identified <5000 protein groups with higher CoV, suggesting an overestimation of 120 ng, probably due to lower tryptic peptide recovery. For an optimal quantitative proteomics analysis, it is desirable to have an equal or similar amount of input material, preferably in the range of 5–10 µg, and for very low input, it is advisable to increase the loading amount for LC-MS to compensate for the lower tryptic peptide recovery.

### 3.2. Plasmid DNA Micro-Spin Column for sTRAP Protocol Has Low CoV—Implications from the LC-MS Study

**Figure 3:**
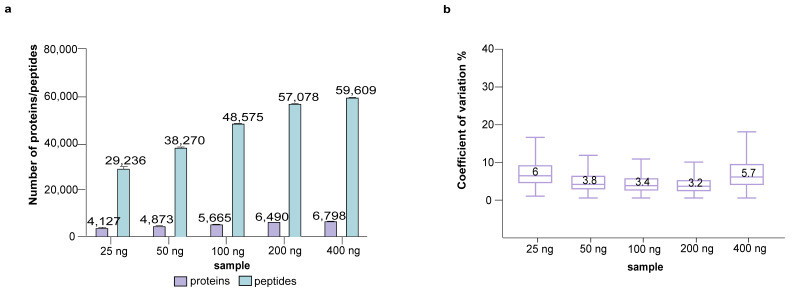
LC-MS analysis of 4 technical replicates per loading concentration with a range of 25–400 ng from a single (10 μg) P2 protein digest prepared by sTRAP protocol. (**a**) Proteins and peptides identified and (**b**) boxplots showing the CoV. X-axis shows the peptide amount used for the LC-MS analysis.

The CoV shown in Figure 2 stems from both the sTRAP sample preparation and the subsequent LC-MS analysis. To assess the variation caused by factors downstream of sTRAP sample preparation, we analyzed four technical replicates of a single P2 sample of 10 μg prepared previously using the sTRAP protocol. This single sample was run in LC-MS multiple times in amounts of 25 ng, 50 ng, 100 ng, 200 ng and 400 ng (Figure 3, Table S1). The stepwise increase from 25 ng to 200 ng resulted in a considerable increase in the number of identified proteins and peptides; doubling the loading amount allowed the identification of about 9000 more peptides. The CoV was around 3.5%, except for the lowest loading of 25 ng with a higher CoV. The overall CoV shown in Figure 2 of about 3.5–4% can therefore be explained largely by the subsequent LC-MS steps (Figure 3, Table S1), demonstrating the high reproducibility of sTRAP sample preparation. High sample loading may saturate the system and negatively affect the quantitation. The use of 400 ng for LC-MS resulted in a lesser increment of protein and peptide identities (Figure 3, Table S1), suggesting that the optimal loading is between 100–200 ng. It should be noted that this optimal loading range is specific to the LC-MS system evaluated here, and that the saturation point will vary between LC-MS systems. Researchers have to identify this within their own system, for example by running a sample loading series.

### 3.3. Comparison of the Sample Preparation Protocol Using Plasmid DNA Micro-Spin Column, the Commercial S-TRAP Column, and In-Gel Digestion Reveals the Favorable Properties of Plasmid DNA Micro-Spin Column for Quantitative Proteomics

**Figure 4:**
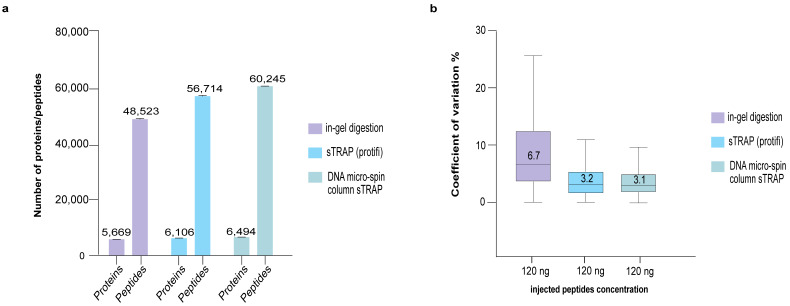
Comparative proteomics analysis of in-gel digestion and sTRAP protocol using commercial S-TRAP column (Protifi) and the plasmid DNA micro-spin column. (**a**) Number of proteins/peptides identified and (**b**) boxplots showing the CoV between the different protocols. The same input sample of 5 μg was used for all three sample preparation protocols, where 120 ng was used for LC-MS with three technical replicates.

To interrogate the validity of the present method, we compared it to the use of a commercial S-TRAP column from Protifi, and the classic in-gel digestion protocol. Figure 4 shows that the use of plasmid DNA micro-spin column resulted in a higher number of identified proteins and peptides and a lower CoV, whereas in-gel digestion protocol yielded a lower number of proteins/peptides and a higher CoV (Table S1). The plasmid DNA micro-spin column appears to perform slightly better than the Protifi column.

### 3.4. Proteomics Analysis of Hippocampi from 5xFAD and Wildtype Mice

**Figure 5:**
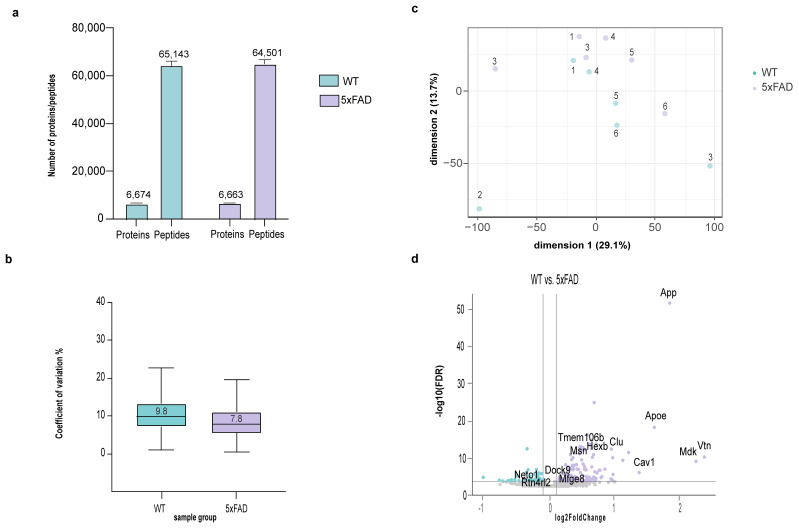
MS-DAP downstream analysis of DIA-NN output from the proteomics data of hippocampal extract from 5xFAD and wildtype mice (six biological replicates per group). (**a**) number of proteins and peptides that were identified per group, (**b**) boxplots showing the CoV within group, (**c**) PCA plot with the two first principal components, explaining sample variance and clustering, (**d**) volcano plot showing the regulated proteins in 5xFAD mice (FDR ≤ 0.05, log2 Fold Change ≤ −0.1 or ≥0.1). Proteins with high absolute log2 fold change and close proximity to APP on STRING analysis (Figure S1) are highlighted.

To demonstrate the applicability of the sTRAP protocol for the expression proteomics experiment with the real-world samples, we examined the brain proteome differences between the 5xFAD and the wildtype mice. An input of 10 µg was processed by sTRAP with the plasmid DNA micro-spin column, of which 120 ng was used for LC-MS/MS. We identified 6600 protein groups, with an average CoV < 9% (Figure 5a,b, Table S1). PCA plot demonstrated the well separation of 5xFAD and the wildtype mice proteomes (Figure 5c). As expected, App is highly regulated in 5xFAD mice. Equally, many AD-associated proteins, including the Clu, ApoE, Hexb, are highly regulated (Figure 5d). Several proteins that have been implicated in AD pathology but are less described in previous proteomics studies, for example Cav1, Mdk, Vtn, are also highlighted, as they have high log2 fold changes between the wildtype and the 5xFAD mice.

**Figure 6:**
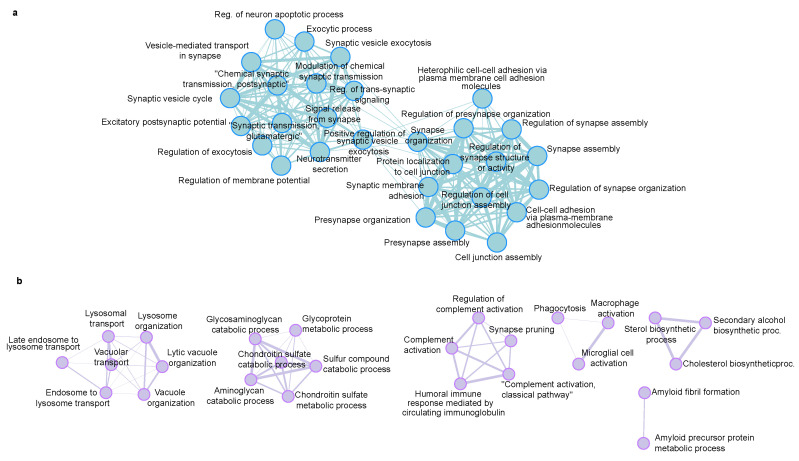
Biological processes GO enrichment analysis of (**a**) down-regulated proteins and (**b**) up-regulated proteins in 5xFAD mice, generated with ShinyGo (set to 30 pathways, minimum pathway size 5, maximum 500).

We used ShinyGO to reveal the enrichment of biological processes that are regulated in the 5xFAD mice. GO terms are in good agreement with the known AD-induced changes. The down-regulated proteins are mainly related to the synaptic processes (Figure 6a), which agrees with the reduction of synapses and neuronal cell death. Similarly, the up-regulated proteins are related to the amyloid precursor protein metabolic process, lysosome, immune effector process and synapse pruning (Figure 6b).

We next used string interaction analysis to reveal the structural and functional interactions of the regulated proteins. In the up-regulated protein set, App is positioned centrally in the protein network (Figure S1). This agrees with the central role of the App abnormal processing in the AD pathology-related protein changes. The other typical risk factors such as Apoe and Clu are indeed highly regulated (Figure 5d, Table S2). The detection of Tmem106b is particularly interesting. Tmem106b is associated with frontotemporal dementia (FTD), but recent GWAS studies also demonstrated its association with AD. It has been argued that the possibility of the misclassification of some FTD as AD cases cannot be excluded, which may cause the misassignment of Tmem106b as a risk factor for AD [24]. Nevertheless, the detection of Tmem106b as a regulated protein in the AD mouse model supports the argument that this protein may be involved in the development of AD. Similarly, STRING protein network analysis of the dysregulated proteins in 5xFAD revealed high proximity and interaction of proteins that are less mentioned in previous AD proteomics studies (Msn, Cav1, Vtn, Mfge8, Gef, Neto1, Rtn4rl2, Fbs1) with App (Figure S1).

## 4. Discussion

In this study, we demonstrate the use of a low-cost plasmid DNA micro-spin column for proteomics sample preparation at low to sub-µg inputs with high reproducibility. We then applied this workflow to the comparative analysis of the hippocampal proteomes from an AD mouse model and its wildtype littermates.

Among the popular sample preparation protocols for proteomics analysis, sTRAP has several advantageous properties; it is a simple and fast protocol, has good reproducibility, and is able to remove a wide range of small molecular contaminants [25]. Here, we demonstrate that the widely commercially available plasmid DNA micro-spin column is an excellent alternative tool for sTRAP as it yields a higher recovery rate of identified proteins and peptides and a lower CoV, when compared with the commercially available Protifi column and the in-gel digestion protocol. Furthermore, it is much cheaper (in our case, it costs about €0.3 per micro-spin column), and easier to use. All reagents are readily available in a typical laboratory. Because of the simplicity of the procedure, this protocol can be performed by simply following the Methods section, with practically no hands-on time. Importantly, this protocol handles input that is most relevant for biological samples of 10 µg to 0.3 µg, with the downstream analysis using 100–200 ng tryptic peptides in a modern LC-MS setup, in this case the Evosep-TimsTOF Pro 2. Higher loading of 50 µg input has also been analyzed successfully with the same protocol (data not shown). Fast trypsin digestion can be performed at 47 °C for 1 h with a higher concentration of trypsin (tryspin/protein ratio of 1/10) (see [8]). Nevertheless, we favor the classic overnight digestion procedure at 37 °C as it is easier to perform, and to avoid the possibility of the column drying under a higher incubation temperature. 

We tested the reproducibility and recovery of samples on sTRAP with a serial dilution of a single sample. Using a loading of 120 ng tryptic peptides to LC-MS from the 10 µg input on the sTRAP column, we obtained a depth of approximately 5700 protein groups with a median CoV of 4%. In the case of the lowest loading of about 300 ng on the micro-spin column and 120 ng tryptic peptide used for LC-MS analysis, we identified 4800 protein groups with a median CoV around 8%. Therefore, for very low input samples, it is advisable to increase the loading amount for LC-MS, as the tryptic peptide recovery is lower.

Proteomics analyses of brains from various mouse models of AD have been reported previously [26,27,28,29]. In the present study, we analyzed the hippocampus from 5xFAD mice using the sTRAP protocol. The up-regulated proteins are largely involved in amyloid protein metabolic process, immune effectors process, glial cells activation and lysosome transport. These are the typical AD associated pathways observed in human AD patients [28,30]. Many of the core proteins identified in the current study have also been described in APP/PS-1 mouse, including the well-described Clu, C1qa/b/c, App, Hexb, Apoe and Clcn6, reflecting the consistent changes induced by Aβ in different AD mouse models. Of interest is the detection of up-regulation of Tmem106b, a lysosomal/endosomal protein that has been of great interest lately, as it has been shown to form amyloid filaments in the aged human brain [31] and, in a similar manner, in different neurodegenerative diseases [32,33,34].

Network analysis further reveals the extensive protein-protein interaction of co-regulated proteins in specific clusters, where App appears to be central (Figure S1). Among the interactors with high proximity, Moesin (Msn) is a protein that was up-regulated in the 5xFAD mice and was observed in microglia in non-demented and AD cells, co-localizing with Aβ in amyloid plaques in AD (late and early onset), as well as in Down syndrome [35,36]. Another up-regulated protein, Cav1, is present in the caveole at the cell membrane leads to memory deficits and oxidative stress related to AD and maintains mitochondrial morphology and function in AD with synapsin-promoted overexpression [37,38]. Compared with healthy aging, endothelial-enriched levels of Cav1 are reduced in type 2 diabetes and negatively correlated with Aβ levels [39]. Victronectin (Vtn) is found to co-localize within Aβ deposits in AD and abnormal deposits associated with other age-related degenerative diseases, through calcium ion-binding and H-X domains [40,41,42], and is also found to be up-regulated in our transgenic mice. Lactadherin (Mfge8) was initially reported to be important in the Aβ 1–42 phagocytosis and its expression to be reduced in AD [43]. In our data, Mfge8 is up-regulated in 5xFAD samples. Mfge8 is shown to co-localize with vascular-amyloid deposits, with AD patients having higher levels and cognitive decline regardless of plaque and tau pathology. It promotes Aβ aggregation by forming heterologous fibrils and thus is suggested as a therapeutic target for vascular damage prevention [44]. It is also observed to be highly associated with CAA pathology and has been proposed as a CSF biomarker [45]. Up-regulated in our disease-like mouse model was also Dedicator of Cytokinesis 9 (Dock9), a guanine nucleotide-exchange factor (Gef) that activates Cell Division Cycle 42 (Cdc42) [46]. This result is in accordance with our previous study on the APP/PS1 Alzheimer mouse model, in which we observed up-regulation at both 6- and 12- month-old mice, suggesting that Dock9 has a role in Aβ production or as an early responder to increased Aβ levels [26]. A known linked node to App, and down-regulated in 5xFAD mice, is Neurophillin and tolloid-like protein 1 (Neto1), which is associated with AMPA receptor but also found to be a component of the NMDA receptor via GluN2A or GluN2B subunits, and required for synaptic plasticity and learning [47]. Neto1 was also reported to regulate endogenous somatodentric kainite receptors in interneurons [48]. In addition, Reticulon 4 Receptor Like 2 (Rtn4rl2) belongs to the Nogo receptor family (NgR), which mediates the inhibition of synaptic plasticity via Aβ. Aβ peptides block the assembly of new synapses through T-Type calcium channels inhibition in a Nogo receptor-mediated manner [49]. Except for App, Rtn4rl2 binds glycoprotein Mag and inhibits the neurite outgrowth and is bound by the F-box protein that recognizes the sugar chain 1 (Fbs1) leading to lower levels of Bace1 and the rescue of synaptic deficits [50,51]. This agrees with the down-regulation of this protein observed in our AD mouse model.

## Data Availability

The mass spectrometry proteomics datasets generated during the current study are available in the PRIDE repository, with the identifier PXD041045.

## Supplementary Materials

The following supporting information can be downloaded at: https://www.mdpi.com/article/10.3390/cells12091242/s1, Figure S1. STRING protein network analysis revealing the interactomes of the dysregulated proteins (the edges indicate both physical and functional protein association); Table S1. Quantification results for all the experiments; Table S2. Dysregulated proteins in 5xFAD mice (log2FC: Log2 Fold Change. FDR: False Discovery Rate).
