# Supplementary material for: Suspension TRAPping Filter (sTRAP) Sample Preparation for Quantitative Proteomics in the Low µg Input Range Using a Plasmid DNA Micro-Spin Column: Analysis of the Hippocampus from the 5xFAD Alzheimer’s Disease Mouse Model
Source: Cells. 2023 Apr 25;12(9):1242. doi: 10.3390/cells12091242 (PMC10177283; doi:10.3390/cells12091242)
Supplement: Supplementary file 1 [file cells-12-01242-s001.zip › Figure S1.pdf]

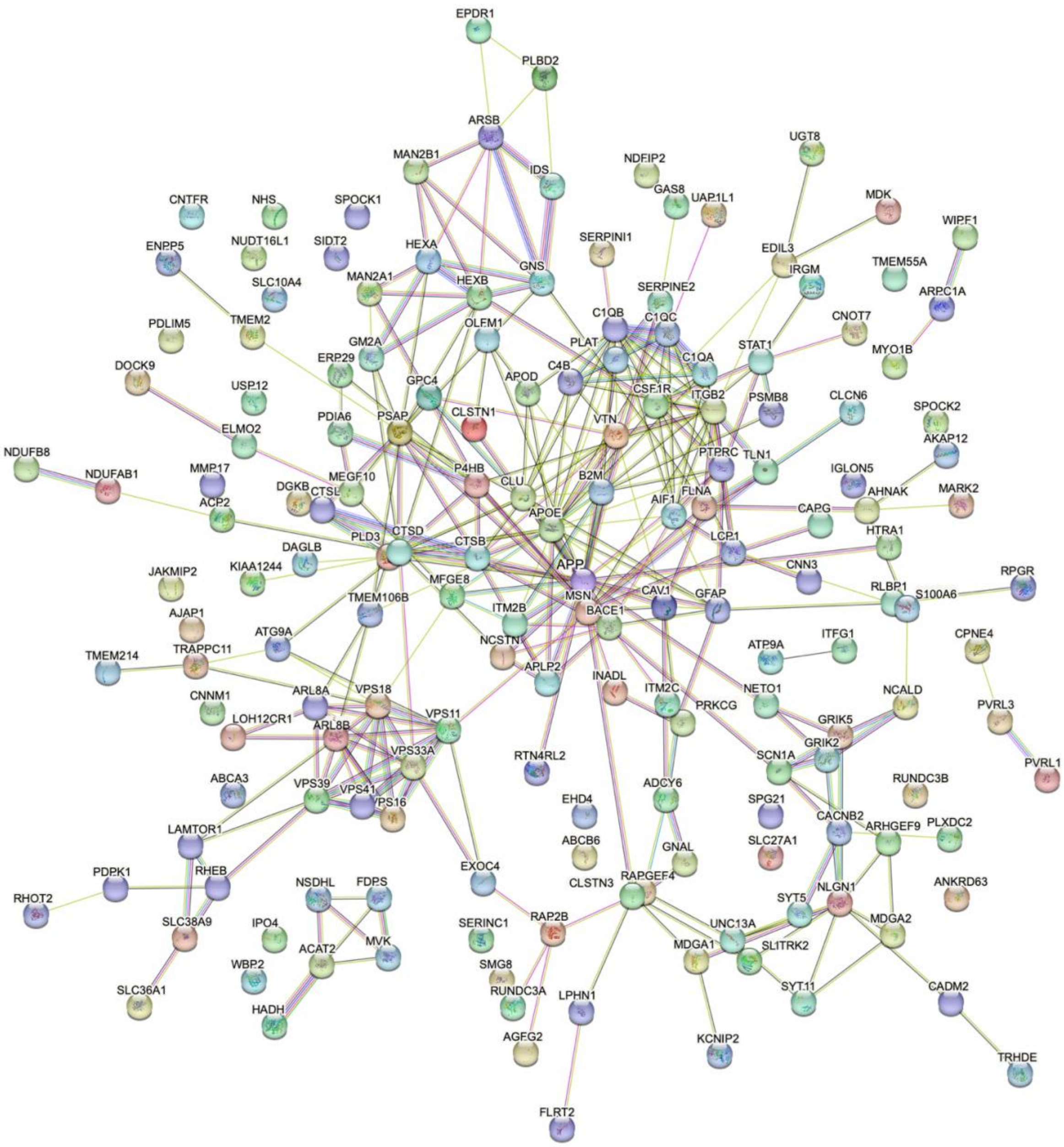

Figure S1. STRING protein network analysis revealing the interactomes of the dysregulated proteins (the edges indicate both physical and functional protein association).
