# Supplementary material for: Suspension TRAPping Filter (sTRAP) Sample Preparation for Quantitative Proteomics in the Low µg Input Range Using a Plasmid DNA Micro-Spin Column: Analysis of the Hippocampus from the 5xFAD Alzheimer’s Disease Mouse Model
Source: Cells. 2023 Apr 25;12(9):1242. doi: 10.3390/cells12091242 (PMC10177283; doi:10.3390/cells12091242)
Supplement: Supplementary file 1 [file cells-12-01242-s001.zip › Table S1.pdf]

Table S1. Quantification results for all the experiments.

Sample	Proteins	Peptides	Group CoV
**Serial Diluted -1.2%**			
0.3µg a	1,860	7,165	9.2
0.3µg b	1,714	6,781	9.2
0.3µg c	1,756	6,854	9.2
0.3µg d	1,678	6,433	9.2
0.6µg a	2,746	12,360	11.3
0.6µg b	2,779	12,152	11.3
0.6µg c	2,702	12,056	11.3
0.6µg d	2,700	11,938	11.3
1.25µg a	3,431	16,263	6
1.25µg b	3,560	17,123	6
1.25µg c	3,490	16,734	6
1.25µg d	3,525	16,771	6
2.5µg a	4,152	22,208	4.3
2.5µg b	4,166	22,254	4.3
2.5µg c	4,159	22,334	4.3
2.5µg d	4,210	22,526	4.3
5µg a	5,106	30,139	3.4
5µg b	5,186	30,440	3.4
5µg c	5,136	30,043	3.4
5µg d	5,192	30,531	3.4
10µg a	6,094	38,773	3.9
10µg b	6,092	39,186	3.9
10µg c	6,124	39,265	3.9
10µg d	6,112	39,448	3.9
**Serial Diluted-120 ng**			
0.3µg a	5,089	26,764	8.1
0.3µg b	5,070	26,893	8.1
0.3µg c	5,087	27,065	8.1
0.3µg d	4,967	25,570	8.1
0.6µg a	5,162	27,804	5.4
0.6µg b	5,135	26,872	5.4
0.6µg c	5,139	27,439	5.4
0.6µg d	5,176	27,664	5.4
1.25µg a	5,437	30,626	5.7
1.25µg b	5,573	31,804	5.7
1.25µg c	5,555	31,671	5.7
1.25µg d	5,556	31,466	5.7
2.5µg a	5,627	33,013	3.8
2.5µg b	5,595	32,937	3.8
2.5µg c	5,644	33,152	3.8
2.5µg d	5,617	32,915	3.8
5µg a	5,866	36,190	3.5
5µg b	5,938	36,760	3.5
5µg c	5,907	36,094	3.5
5µg d	5,920	36,507	3.5

Sample	Proteins	Peptides	Group CoV
10µg a	5,971	37,457	3.6
10µg b	5,981	37,972	3.6
10µg c	6,021	38,090	3.6
10µg d	6,011	38,271	3.6
**LC-MS implications test**			
25 ng a	4,203	24,165	6
25 ng b	4,128	24,233	6
25 ng c	4,230	23,489	6
50 ng a	4,183	34,656	3.8
50 ng b	5,292	34,617	3.8
50 ng c	5,280	34,490	3.8
100 ng a	6,175	45,625	3.4
100 ng b	6,161	45,621	3.4
100 ng c	6,163	45,581	3.4
200 ng a	6,696	52,190	3.2
200 ng b	6,696	51,696	3.2
200 ng c	6,686	52,090	3.2
400 ng a	6,923	52,694	5.7
400 ng b	6,931	52,760	5.7
400 ng c	6,909	52,659	5.7
**In-gel/sTRAP protifi/sTRAP DNA micro-spin column**			
Gel a	5,683	48,574	6.7
Gel b	5,686	48,753	6.7
Gel c	5,638	48,242	6.7
Protifi 1	6,127	56,913	3.2
Protifi 2	6,121	56,822	3.2
Protifi 3	6,071	56,408	3.2
DNA column 1	6,462	60,255	3.1
DNA column 2	6,495	60,326	3.1
DNA column 3	6,525	60,153	3.1
**Animal study WT vs 5xFAD**			
WT 1	7,357	61,688	9.8
WT 2	7,135	57,748	9.8
WT 3	7,246	61,078	9.8
WT 4	7,416	62,949	9.8
WT 5	7,405	62,862	9.8
WT 6	7,395	62,249	9.8
5xFAD 1	7,402	63,077	7.8
5xFAD 2	7,381	61,783	7.8
5xFAD 3	7,400	62,368	7.8
5xFAD 4	7,205	58,837	7.8
5xFAD 5	7,410	62,776	7.8
5xFAD 6	7,455	63,471	7.8
