# Supplementary material for: Suspension TRAPping Filter (sTRAP) Sample Preparation for Quantitative Proteomics in the Low µg Input Range Using a Plasmid DNA Micro-Spin Column: Analysis of the Hippocampus from the 5xFAD Alzheimer’s Disease Mouse Model
Source: Cells. 2023 Apr 25;12(9):1242. doi: 10.3390/cells12091242 (PMC10177283; doi:10.3390/cells12091242)
Supplement: Supplementary file 1 [file cells-12-01242-s001.zip › Table S2.pdf]

Table S2. Dysregulated proteins in 5xFAD mice (log2FC: Log2 Fold Change. FDR: False Discovery Rate).

Gene symbol	log2FC	FDR	Gene symbol	log2FC	FDR	Gene symbol	log2FC	FDR	Gene symbol	log2FC	FDR
Up-regulated proteins									Down-regulated proteins		
VTN	2.33	0.00	NDFIP2	0.54	0.05	BACE1	0.31	0.01	CNNM1	-0.17	0.00
MDK	2.20	0.00	LCP1	0.52	0.00	VPS11	0.31	0.00	ELMO2	-0.18	0.00
APP	1.80	0.00	ENPP5	0.52	0.00	EDIL3	0.30	0.00	NCALD	-0.18	0.02
APOE	1.57	0.00	MVK	0.52	0.00	TMEM214	0.30	0.03	MMP17	-0.19	0.04
CAV1	1.35	0.00	GNS	0.52	0.00	UGT8	0.30	0.03	SCN1A	-0.19	0.05
H2-L	1.19	0.00	MAN2B1	0.50	0.00	LAMTOR1	0.28	0.00	CACNB2	-0.19	0.01
CAPG	1.10	0.00	PSMB8	0.50	0.04	JAKMIP2	0.28	0.00	RAP2B	-0.20	0.00
C1QC	0.95	0.00	ARL8A	0.50	0.00	VPS18	0.28	0.00	ARHGEF9	-0.20	0.00
LYZ2	0.95	0.04	TMEM106B	0.50	0.00	CNN3	0.28	0.03	OLFM1	-0.20	0.00
C4B	0.94	0.00	HEXA	0.49	0.00	APLP2	0.27	0.00	RHOT2	-0.21	0.00
H2-K1	0.93	0.02	CLSTN3	0.48	0.00	MFGE8	0.27	0.04	SLC27A1	-0.21	0.02
CLU	0.93	0.00	NUDT16L1	0.47	0.04	RLBP1	0.27	0.02	CPNE4	-0.21	0.03
SIDT2	0.92	0.01	NSDHL	0.47	0.02	CTSL	0.26	0.04	AGFG2	-0.21	0.04
C1QB	0.83	0.00	PLBD2	0.46	0.00	AKAP12	0.25	0.03	CADM2	-0.21	0.01
1700006A1RIK	0.78	0.00	RPGR	0.46	0.04	PDLIM5	0.24	0.03	NLGN1	-0.21	0.01
C1QA	0.77	0.01	CSF1R	0.46	0.00	RUNDC3A	0.24	0.00	SLITRK2	-0.22	0.03
HTRA1	0.75	0.00	PLD3	0.46	0.00	TLN1	0.24	0.00	GRIK2	-0.23	0.03
B2M	0.74	0.00	ITGB2	0.46	0.01	CLSTN1	0.23	0.03	NECTIN1	-0.23	0.00
STAT1	0.71	0.00	ARL8B	0.45	0.00	VPS16	0.23	0.00	NDUFAB1	-0.23	0.03
HEXB	0.70	0.00	MSN	0.44	0.00	RHEB	0.22	0.02	MDGA2	-0.24	0.04
MAN2A1	0.69	0.01	SPOCK1	0.43	0.00	UAP1L1	0.22	0.04	NETO1	-0.24	0.04
PTPRC	0.69	0.04	ATG9A	0.41	0.03	ITFG1	0.21	0.02	IGLON5	-0.25	0.05
SLC36A1	0.68	0.04	GM2A	0.41	0.01	PDIA6	0.20	0.04	CNTFR	-0.26	0.03
GFAP	0.67	0.00	FLNA	0.41	0.00	SERPINE2	0.20	0.04	NDUFB8	-0.28	0.02
CLCN6	0.67	0.00	EPDR1	0.41	0.02	P4HB	0.20	0.00	GPC4	-0.28	0.02
IRGM1	0.66	0.00	SERINC1	0.40	0.00	BORCS5	0.19	0.01	FLRT2	-0.29	0.03
ITM2B	0.66	0.00	PLXDC2	0.39	0.01	EHD4	0.17	0.01	ANKRD63	-0.30	0.00
MYO1B	0.64	0.01	CTSD	0.38	0.00	HADH	0.17	0.04	GNAL	-0.30	0.03
FDPS	0.64	0.00	ARSB	0.38	0.00	TRAPPC11	0.17	0.02	ADCY6	-0.31	0.03
DAGLB	0.63	0.00	ACAT2	0.38	0.00	WBP2	0.16	0.02	MDGA1	-0.33	0.00
SYT11	0.63	0.00	PIP4P2	0.38	0.04	DOCK9	0.14	0.00	DGKB	-0.34	0.00
SPOCK2	0.62	0.00	AIF1	0.37	0.04	ERP29	0.14	0.01	NECTIN3	-0.36	0.00
WIPF1	0.62	0.04	CTSB	0.36	0.00	VPS33A	0.13	0.04	NHS	-0.36	0.02
ABCA3	0.62	0.00	SERPINI1	0.36	0.00	Down-regulated proteins			GRIK5	-0.41	0.04
S100A6	0.60	0.02	ABCB6	0.36	0.01	MARK2	-0.10	0.02	CNOT7	-0.47	0.04
ITM2C	0.60	0.00	ATP9A	0.35	0.00	EXOC4	-0.13	0.00	KCNIP2	-0.50	0.00
APOD	0.59	0.00	VPS39	0.35	0.01	PRKCG	-0.14	0.00	CEMIP2	-0.53	0.02
NCSTN	0.57	0.00	SYT5	0.35	0.00	RAPGEF4	-0.15	0.02	AJAP1	-0.58	0.04
AHNAK	0.57	0.01	ACP2	0.34	0.00	ARPC1A	-0.15	0.04	MEGF10	-0.58	0.02
GM1673	0.57	0.03	PSAP	0.34	0.00	PDPK1	-0.15	0.01	PATJ	-0.60	0.03
SLC38A9	0.56	0.04	ARFGEF3	0.33	0.00	RTN4RL2	-0.16	0.05	PLAT	-0.63	0.04
IDS	0.55	0.00	VPS41	0.33	0.00	TRHDE	-0.16	0.04	SLC10A4	-0.71	0.04
SPG21	0.54	0.00	IPO4	0.32	0.00	ADGRL1	-0.16	0.05	USP12	-0.76	0.03
GAS8	0.54	0.03	RUNDC3B	0.32	0.02	UNC13A	-0.17	0.00	SMG8	-1.01	0.00
